# Halo-RPD: searching for RNA-binding protein targets in plants

**DOI:** 10.18699/vjgb-24-09

**Published:** 2024-02

**Authors:** A.O. Shamustakimova

**Affiliations:** All-Russian Research Institute of Agricultural Biotechnology, Moscow, Russia

**Keywords:** A. thaliana, HaloTag, RNA-binding proteins, RNA pulldown assay, RNA-protein complexes, cold-shock domain protein, A. thaliana, HaloTag, РНК-связывающие белки, соосаждение, РНК, РНК-белковые комплексы, белок с доменом холодового шока

## Abstract

Study of RNA-protein interactions and identification of RNA targets are among the key aspects of understanding RNA biology. Currently, various methods are available to investigate these interactions with, RNA immunoprecipitation (RIP) being the most common. The search for RNA targets has largely been conducted using antibodies to an endogenous protein or to GFP-tag directly. Having to be dependent on the expression level of the target protein and having to spend time selecting highly specific antibodies make immunoprecipitation complicated. Expression of the GFP-fused protein can lead to cytotoxicity and, consequently, to improper recognition or degradation of the chimeric protein. Over the past few years, multifunctional tags have been developed. SNAP-tag and HaloTag allow the target protein to be studied from different perspectives. Labeling of the fusion protein with custom-made fluorescent dyes makes it possible to study protein expression and to localize it in the cell or the whole organism. A high-affinity substrate has been created to allow covalent binding by chimeric proteins, minimizing protein loss during protein isolation. In this paper, a HaloTag-based method, which we called Halo-RPD (HaloTag RNA PullDown), is presented. The proposed protocol uses plants with stable fusion protein expression and Magne® HaloTag® magnetic beads to capture RNA-protein complexes directly from the cytoplasmic lysate of transgenic Arabidopsis thaliana plants. The key stages described in the paper are as follows: (1) preparation of the magnetic beads; (2) tissue homogenization and collection of control samples; (3) precipitation and wash of RNA-protein complexes; (4) evaluation of protein binding efficiency; (5) RNA isolation; (6) analysis of the RNA obtained. Recommendations for better NGS assay designs are provided.

## Abstract (ru)

Изучение РНК-белковых взаимодействий и идентификация РНК-мишеней относятся к важнейшим аспектам понимания биологии РНК. К настоящему времени предложены различные методы изучения таких взаимодействий; одним из широко распространенных является иммунопреципитация РНК (RIP). Большинство работ по поиску РНК-мишеней было проведено с использованием антител непосредственно на эндогенный белок или же на GFP, слитый с целевым белком. Зависимость от уровня экспрессии целевого белка и подбор специфичных антител значительно затрудняют классическую иммунопреципитацию. Белок же, слитый с GFP, нередко может быть цитотоксичен, что в дальнейшем приведет к его неправильному узнаванию и/или деградации. В последние годы был разработан ряд мультифункциональных тагов, включая SNAP-tag и HaloTag. Такие таги способствуют изучению целевых белков с разных сторон. Для них созданы флуоресцентные красители, способные прочно связываться с определенным участком тага. Это позволяет как изучать наработку химерного белка, так и определять его локализацию непосредственно в клетке или во всем организме. Для таких тагов разработаны также высокоаффинные субстраты, ковалентно связывающие химерные белки, что значительно сокращает потери в ходе выделения. В данной работе представлен метод, основанный на системе HaloTag, который мы назвали Halo-RPD (HaloTag RNA PullDown). В протоколе используются растения со стабильной экспрессией химерного белка и магнитные шарики Magne® HaloTag® Beads для захвата РНК-белковых комплексов непосредственно из цитоплазматического лизата трансгенных растений Arabidopsis thaliana. Приводится описание основных этапов: 1) подготовка магнитных шариков; 2) гомогенизация тканей и отбор контролей; 3) осаждение и отмывка РНК-белковых комплексов; 4) определение эффективности связывания белка; 5) выделение РНК; 6) анализ полученной РНК. Даны рекомендации для планирования эксперимента по высокопроизводительному секвенированию.

## Introduction

RNA-binding proteins play a major part in complex cellular processes, such as differentiation, development, responses to biotic and abiotic stress factors, and post-transcriptional control

In recent years, the variety of methods for studying RNAprotein interactions has expanded significantly (Ramanathan et al., 2019). However, some older technologies, such as RNA-immunoprecipitation (RIP), still remain rather common (Brooks, Rigby, 2000). The latter is based on in vivo mapping of RNA-protein interactions using crosslinking agents, such as ultraviolet or formaldehyde. Currently, RIP is used in the vast majority of studies investigating RNA-protein complexes in plants (Köster, Meyer, 2018; Frydrych Capelari et al., 2019; Seo, Chua, 2019; Steffen et al., 2019)

Despite its wide use, RIP has several downsides, e. g. UV radiation induces the formation of the irreversible covalent bond between a protein and RNA; formaldehyde not only binds the protein of interest to RNA but crosslinks its partner proteins as well; highly specific antibodies are required for a successful outcome

HaloTag-fused proteins are used in the proposed protocol as an alternative to RIP (Los et al., 2008). Initially, the HaloTag technology was intended and successfully used for precipitation of protein-protein and DNA-protein complexes from bacterial and mammalian cell lysate (Urh et al., 2008). However, in the last few years, the technology was adapted (van Dijk et al., 2015; Banks et al., 2016; Li et al., 2020) and modified (Gu et al., 2018) to identify RNA-protein complexes in tissue cells in humans and animals

So far, there have been only two papers, the authors of which investigated the use of HaloTag technology for the search of partner proteins in plants (Samanta, Thakur, 2017; Ren et al., 2020). In the first paper, the authors analyzed mediator proteins in transgenic plants of rice, and in the second one, the binding site of transcription factor ZmNST3 in transgenic plants of maize was investigated.

The goal of the present study was to design a protocol for isolating RNA-protein complexes from the cytoplasm of Arabidopsis thaliana plants using the HaloTag technology. Papers (Sorenson, Bailey-Serres, 2015; Banks et al., 2016) were used as a reference to design the protocol called HaloTag RNA-PullDown (Halo-RPD).

## The Halo-RPD method

Plant material. Two lines of Arabidopsis thaliana (L.) Heynh. Columbia ecotype were used in the study as transgenic plants with stable expression of HaloTag and EsCSDP3-HaloTagfused proteins (Taranov et al., 2018).

**Fig. 1.**
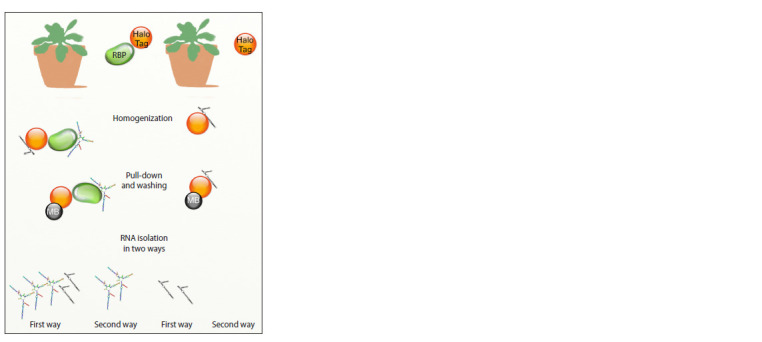
A simplified Halo-RPD assay design. Transgenic plants expressing HaloTag-fused RNA-binding protein (on the left) and plants expressing the HaloTag protein (on the right). Plant tissue is homogenized to obtain cytoplasmic lysate and incubated with the magnetic beads capable of binding the HaloTag protein by covalent bonds. The beads with precipitated target protein complexes are washed, and RNA is isolated in two ways. The first way is to isolate RNA directly from the magnetic beads by incubation in the ExtractRNA reagent, and the second implies elution in TEV buffer and further isolation using the ExtractRNA reagent.

The stages of HaloRPD protocol for isolation of RNA-protein complexes are as follows: preparation of magnetic beads; tissue homogenization, and collection of control samples; pull-down and wash of RNA-protein complexes; evaluation of protein binding efficiency; RNA isolation; analysis of the obtained RNA. A simplified assay design is presented in Figure 1.

Preparation of magnetic beads

The Magne HaloTag Beads (Promega Corp.) magnetic particle suspension (100 μl) was placed in two tubes for the experimental (EsCSDP3-HaloTag) and control (HaloTag) samples. The tubes were incubated on a magnetic stand until transparent, and the liquid was carefully removed without disturbing the beads. The buffer (400 μl) composed of 50 mM Tris-HCl (pH 7.4), 137 mM NaCl, 2.7 mM KCl, and 0.05 % Igepal Ca- 630 (Promega Corp.) was added to the beads, and the suspension was gently mixed manually several times. The tubes were transferred to a magnetic stand, incubated until transparent, and liquid was fully removed by pipetting. The tubes were then washed two more times. The supernatant was not removed after the third wash. The tubes were stored at +4 °С.

The manufacturer offers two types of substrate for pulldown of fusion protein from lysate, namely HaloLink resin (Promega Corp.) and MagneBeads (Promega Corp.), the latter being a newer product. The magnetic beads have an advantage of high binding affinity of HaloTag-fused proteins and low non-specific binding level. For instance, 1 ml of magnetic beads binds over 20 mg of protein, whereas the same amount of resin only binds 7 mg. The resin was used in prior assays of our study, which significantly increased their duration due to multiple centrifugation steps. This approach also required the availability of LowBind tubes or silicone treatment of the tubes to minimize resin loss.

Tissue homogenization and collection of control samples

Tissue homogenization and collection of control samples For better preservation of plant tissue, leaf blades were wrapped in foil and placed in liquid nitrogen

Precooled mortars and pestles were used for homogenization. The tissue was placed in a mortar, a small amount of liquid nitrogen was added, and the mixture was homogenized to a powder. The obtained powder was then added to a tube with 300 μl of lysis buffer composed of 50 mM Tris-HCl (pH 7.5), 150 mM NaCl, 1 % Triton X-100, 0.1 mM benzamidine HCl, 55 μM phenanthroline, 10 μM bestatin, 20 μM leupeptin, 5 μM pepstatin A, 1 mM PMSF, 1 mM DTT, and 3 μl RiboLock™ (Thermo Fisher Scientific). The tubes were then sealed, mixed on a vortex mixer, and put on ice to cool down. The obtained homogenate was centrifuged at +4 °С for 7 minutes at maximum speed. The supernatant was carefully transferred to clean precooled tubes without disturbing the debris. The obtained lysate had a rather high detergent content, which could potentially cause dissociation of RNA-protein complexes and weaken the protein’s bond with the substrate. To avoid this, 700 μl of the buffer composed of 50 mM Tris- HCl (pH 7.4), 137 mM NaCl, 2.7 mM KCl was added to the lysate. At this stage, two control samples were collected: (1) lysate samples of 100 μl were placed in separate tubes for future analysis of RNA input fraction; (2) lysate samples of 10 μl were placed in 0.6 μl tubes to evaluate the binding efficacy between the target protein and the substrate. Both types of tubes were stored at +4 °С.

Pull-down and wash of RNA-protein complexes

The tubes with the magnetic beads prepared earlier were placed on a magnetic stand, and the excess liquid was removed. The tubes were then removed from the stand, and the obtained diluted lysate was added to the beads. The tubes were placed on an orbital shaker, incubated at constant rotation for two hours at +4 °С, and transferred back to the magnetic stand. Lysate samples of 10 μl were collected to control the protein’s bond with the substrate, the remaining liquid was carefully removed.

The magnetic beads were washed by adding 400 μl of the buffer composed of 50 mM Tris-HCl (pH 7.4), 137 mM NaCl, 2.7 mM KCl, and 0.05 % Igepal Ca-630 (Promega Corp.). The tubes were gently shaken manually three times and placed on the magnetic stand. It should be noted that the number of washes is chosen for each RNA-protein complex on an individual basis. The assay described here included five washes. The tubes were incubated on an orbital shaker for five minutes at +4 °С during the last wash.

When the last wash was finished, the tubes were put on ice

Evaluation of binding efficiency between protein and magnetic beads

At this stage, content, preservation, and binding efficiency of the target protein were evaluated. For this purpose, tubes with the previously collected lysate fractions described in sections “Tissue homogenization and collection of control samples” and “Pull-down and wash of RNA-protein complexes” were further analyzed. 1 μl of 50 μM HaloTag® TMR Ligand (Promega Corp.) was added to each tube. The content was mixed by pipetting, and the tubes were kept in the dark for 15 minutes. Then, 10 μl of 4× SDS loading buffer was added and the mixture was heated for 2 minutes at +90 °C. We prepared 8 % polyacrylamide gel for Laemmli electrophoresis (Laemmli, 1970) and placed 5 μl of the obtained product in gel wells. The gel was analyzed using a densitometric scanner Typhoon FLA 9000 (GE Healthcare) at the given wavelength (extinction wavelength of 532 nm and emission wavelength of 580 nm).

**Fig. 2.**
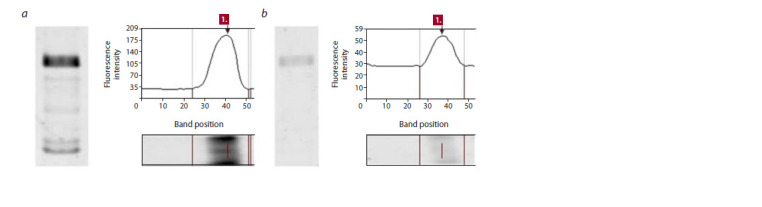
Evaluation of the binding efficiency of the EsCSDP3 protein on magnetic beads using the TMRligand fluorescent dye. a, The lysate sample before binding and the fluorescence level of the bound dye; b, the lysate sample 2 hours after incubation at +4 °С and the fluorescence level of the bound dye. The unbound protein fraction is about 25 %.

It can be seen from Figure 2 that a higher percentage of protein turned out to be bound and therefore was not detectable in the supernatant after 2-hour incubation. Otherwise, the go-to solution is to increase incubation time. A larger amount of magnetic beads increases non-specific binding.

RNA isolation

At this stage, two methods of isolating RNA from the protein complex are available, and prior knowledge of the studied protein, as well as the further course of analysis of the obtained RNA are to be taken into account

For instance, isolating RNA directly from magnetic beads by incubation in the ExtractRNA reagent (Evrogen) (the first method) produces the eluate, which, in addition to the target RNA obtained directly from the protein, includes several nonspecifically bound RNA molecules from HaloTag® and the substrate. This isolation method is preferable, if the further analysis includes RT-PCR or Real-time PCR with primers on the known RNA targets

If NGS is used to identify the nature of unknown RNA targets, then elution in TEV buffer and further extraction using the ExtractRNA reagent (Evrogen) (the second method) is recommended. TEV protease treatment of the RNA-protein complex facilitates its release into the solution, while non-specifically bound RNA molecules stay at the bottom of the tube

To isolate RNA from the eluate, 100 μl of the buffer composed of 50 mM Tris-HCl (pH 8.0), 0.5 mM EDTA, 0.005 mM DTT, 40 U of RiboLock™ (Thermo Fisher Scientific) and 5 U of HaloTEV protease (Promega Corp.) was added to the washed beads with the precipitated target RNA-protein complex. The tube was placed on an orbital mixer and incubated overnight at +4 °С. The next day, the tubes were placed on a magnetic stand, and 90 μl of the eluate was transferred to a clean 1.5 ml tube. Then, 1 ml of the ExtractRNA reagent (Evrogen) was added to the obtained eluate. At this stage, RNA isolation from the beads and from the eluate proceeded identically. We similarly added 1 ml of the ExtractRNA reagent (Evrogen) to the tubes with magnetic beads washed in the buffer solution, and incubated the tubes on a magnetic stand at room temperature for 5 minutes with careful intermittent mixing. Then 200 μl of chloroform was added and the content was mixed on a vortex mixer for 30 seconds. The tubes were then centrifuged at +4 °С at 10,000g for 10 minutes. We carefully collected 500 μl of the aqueous phase and transferred it to a new tube. We then added 25 μg of glycogen, mixed the content by pipetting, and incubated the tube for 10 minutes at room temperature. The tubes were centrifuged for 10 minutes at 18,000g at room temperature. The supernatant was carefully removed with a small amount of isopropanol left at the bottom, and 1 ml of 75 % ethanol was added to the precipitate. The tubes were incubated at –20 °С overnight. Then the tubes were centrifuged at maximum speed at room temperature for 5 minutes. The supernatant was carefully removed, and the precipitate was dried for 10 minutes at room temperature and eluted into 20 μl of RNase-free water.

Analysis of the obtained RNA

To measure the concentration of the obtained RNA, a Quantus Fluorometer (Promega Corp.) was used. RNA profile was analyzed using a 2100 Bioanalyzer with RNA 6000 Nano and Pico kits (Agilent). Due to the high sensitivity of the device, a sample volume of 1 μl was sufficient for analysis, and therefore a sufficient amount of eluate may be preserved for further experiments

**Fig. 3.**
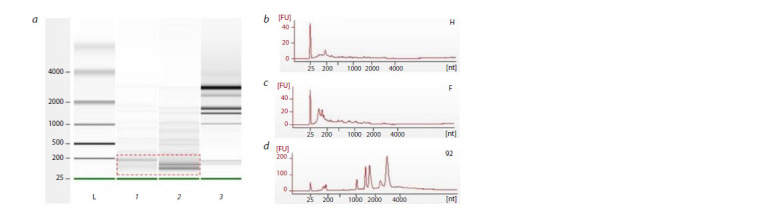
RNA profile obtained using an Agilent 2100 Bioanalyzer with RNA 6000 Nano kit: a–c, RNA is isolated using the first method, where a1 and b correspond to the HaloTag protein sample; a2 and c, to the sample of the EsCSDP3 protein; a3 and d, to the total RNA sample used for reference A dashed outline indicates a small RNA zone. The RNA size compared to the reference marker is measured along the x-axis. The fluorescence intensity of the intercalating dye is measured along the y-axis.

The RNA profile obtained using the 2100 Bioanalyzer is presented in Figure 3. RNA obtained by elution from TEV buffer and further isolation using the ExtractRNA reagent (the second method) is shown in Figure 3, a. Comparison of the two RNA profiles shows that a much wider variety of various RNAs with higher RNA concentration was obtained by elution in the case of RNA-protein complex (plot 1), than in the case of HaloTag (plot 2). After more detailed consideration, we were able to notice that both samples had some common RNAs, and this should be taken into account in further analysis and comparisons

**Fig. 4.**
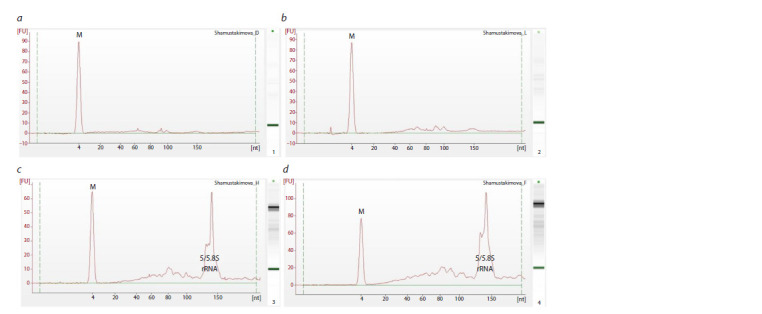
RNA profile obtained using an Agilent 2100 Bioanalyzer with Small RNA kit for small RNA analysis: a and b are the HaloTag and EsCSDP3 protein samples from the assay showed in Figure 3, а (dashed outline); c and d show the RNA isolated from the HaloTag and EsCSDP3 protein samples using the second method The fluorescence intensity of the intercalating dye is measured along the y-axis. The size of the RNA is measured along the x-axis. M – lower alignment marker.

The small RNA zone in samples from Figure 3, a (dashed outline) is shown in Figure 4. This zone was of special interest, since major differences between the samples were found. Its analysis also made it possible to predict the nature of RNA targets. The small RNA zone may be divided as follows: miRNA at nucleotide counts of ~40 and below; transfer RNAs at nucleotide counts of ~40 to ~80; small nuclear/nucleolar and ribosomal RNAs at nucleotide counts of ~80 to ~150.

As expected, comparison of the RNA profiles obtained using two different extraction methods showed a much higher fluorescence intensity in samples obtained using the first method (magnetic beads). In these samples, 5/5.8S ribosomal RNA made up the highest proportion of all RNA types. Comparison of the total fluorescence of the HaloTag and EsCSDP3 samples showed that the signal of the experimental sample (110 FU) was almost twice as intense as that of the control sample (65 FU).

## Preparation of cDNA libraries and sequencing

The proposed protocol does not include a detailed description of cDNA library preparation and further sequencing procedures. It only lists the aspects to be taken into account in assay designs

A cDNA library preparation kit should be selected based on the amount of RNA obtained. Although the A. thaliana genome is relatively short, its number of genes is comparable to that of humans, specifically 27,000 against 25,000. Thus, the recommended read depth is 15–30 million unpaired reads with lengths of 50 bp (Xing et al., 2015; Petri, Jakobsson, 2018). This read length is sufficient for mapping onto the genome, and the given read depth should be sufficient to identify specific targets based on their abundance quantitation in a statistically valid manner.

When RNA is obtained directly from magnetic beads, it is worth using a ribosomal RNA removal kit, since ribosomal RNA would account for a large number of reads due to their high abundance

## Conclusion

The obtained results have shown that the proposed method makes it possible to isolate complexes of fusion proteins and RNA targets from the A. thaliana leaves

The Halo-RPD method has a number of advantages compared to the protocols based on immunoprecipitation, in particular, stable protein expression makes it possible to minimize both the initial amount of plant material for the assay and reagent consumption. The use of the reagent at the stage of RNA isolation from the eluate/substrate allows one to obtain and analyze small RNA and miRNA. The absence of covalent crosslinks and removal of the ultrasonic fragmentation stage make the proposed protocol applicable for analyzing native RNA profiles and thereby drawing preliminary conclusions on the nature of RNA targets, while the use of fluorescent dyes covalently bound to the HaloTag protein makes it possible to control the proper implementation of homogenization and protein isolation stages.

## Conflict of interest

The authors declare no conflict of interest.
